# 

**Published:** 1841-12

**Authors:** 


### THE WESTERN JOURNAL OF MEDICINE AND SURGERY:

EDITED BY

DANIEL DRAKE, M. D.

AND

LUNSFORD P. YANDELL, M. D.

PROFESSORS IN THE LOUISVILLE MEDICAL INSTITUTE.

NO. XXIV.—DECEMBER, 1841.

VOL. IV.—NO. VI.

LOUISVILLE, KY.

PUBLISHED MONTHLY, BY PRENTICE & WEISSINGER,

PROPRIETORS AND PRINTERS.

1841.

*This Number contains 4 sheets—Postage under 100 miles 6 cents, over 100 miles 10 cents.*

